# Practical Medicine and Pathology

**Published:** 1875-12

**Authors:** 


### III. Practical Medicine and Pathology.

1. *The Treatment of Different Forms of Headache.* W. H. Day, M.D. (*Lancet*, Sept., 1875.)

This is an article called forth by a previous one on the same subject by Dr. Moxon. Dr. D. objects to the classification of sick-headache, as depending on the digestive system. "The nervous system is primarily at fault," and the digestive disturbance is a consequence. In persons predisposed to it, overwork, fatigue, late hours and excitement bring it on when no derangement of the digestive organs exists. Some young women with dysmenorrhœa suffer at every menstrual period. The author favors the use of bromide of ammonium and iron tonics, taken for a long time, to modify the nervous sensibility.

The headache occurring in many children who are debilitated, who have some hereditary cachexia, in those who show an aptitude for learning, and who after any mental effort become listless and variable in temper and manner, sometimes dull, at others fidgety and quarrelsome, with weak pulse and frequent sighing and yawning and poor appetite—this headache he thinks is due to some "intricate change in the cerebral membranes or tissues of the brain." The headache may last for months together, and is aggravated by rigid discipline, moral or physical.

Bromide of potassium is the remedy, and must be continued for a long time, and be followed by cod liver oil and other tonics; iodide of potassium is often a valuable addition. He agrees with Dr. Moxon, that when, with headache, sickness of stomach and prostration are extreme, the extremities are cold and pulse feeble, and there is intolerance of light and sound, and the patient has been for days without relief, it is proper to inject, hypodermically, one-sixth of a grain of acetate of morphia. This is often followed by perfect relief. The addition of the 120th or the 80th of a grain of atropia may counteract the tendency to nausea which morphia alone sometimes excites.

2. *Cerebral Rheumatism.* M. BOUCHUT. (*Lyon Médicale; N. Y. Med. Jour.*, Oct., 1875.)

B. stated that both pathological anatomy and the ophthalmoscope proved that the cerebral complication in acute articular rheumatism was a form of meningitis. Examinations of the membranes had shown venous stasis with an opaline infiltration of the pia mater, caused by leucocytes. The ophthalmoscope revealed infiltration of the optic papilla and retina, with dilatation of the retinal veins. In three cases, ushered in by delirium, succeeded by coma and asphyxia, recovery followed the administration of hydrate of chloral, by the mouth, in doses of 45 to 90 grains, and repeated until the delirium had disappeared.

3. *Hystero-Epilepsy Relieved by Pressure of the Ovaries.* CHARCOT. (*Gaz. Méd. de Paris.*)

C. thinks that most hysterical seizures are preceded by an aura starting from one or both ovaries and finds that pressure upon the latter immediately relieves the symptoms. The action was illustrated upon a patient in the Salpétrière affected with hystero-epilepsy, the seizure recurring when the pressure was removed.

He recommends a tourniquet to be used when it is desirable to prolong the pressure, the instrument being applied in the situation and direction in which it is used to compress the iliac artery.

4. *Hysteria in a Male Cured by Compression of the Testicles.* FOET. (*Gaz. Hebdom.*)

The case here reported was one of well marked hysteria in a male patient. It was effectually relieved in less than a minute by compression of the testicles. Antispasmodics were then prescribed, and the man returned next day to his work.

5. *Hystero-Epilepsy with Anuria.* M. BOURNEVILLE. (*London Med. Record; Monthly Abstract.*)

A woman, aet. 23, had always enjoyed good health. Then, in 1854, she was suddenly frightened. The next year, in an attack of hystero-epilepsy she fell into the fire and burnt her face. In 1859–60–63–65 respectively, she had similar crises. In 1866 she had cholera, after which she had suppression of urine for a week. The secretion reappeared, but catheterization became necessary, and was performed daily until 1875. After 1869, she had several attacks of hystero-epilepsy, followed by contractions in the upper and lower limbs. These contractions decreased at times and were partially cured, but recurred and remained permanent in several parts, (the upper and lower limbs of the left side in particular). On May 17, 1875, she had a hystero-epileptic attack preceded by an aura (ovarian and anal pains with irradiations to the epigastrium, neck and temples); there were cries, deviation of the eyes, distortion of the face (which was of a violet tinge); the right arm was bent and remained fixed for three hours. Next day there was contraction of the legs and arms, complete anæsthesia, double amblyopia and contraction of the jaw. The patient could not speak, and there was neuralgia, for which morphine was injected. This condition continued until May 22, when there was an exacerbation, and suddenly the patient was completely cured.

Thus disappeared in a few minutes a retention of urine that had lasted since 1866; a contracture of the jaws, necessitating alimentation by a sound during ten months, aphonia of equal duration, and a contracture of the members of the left side dating from 1869.

The urine had been analyzed frequently. During three months the amount of urine passed was from 230 to 300 grains (Troy) daily, and the amount of urea voided during the same time was from 4.62 to 6.16 grs. (Troy) daily.

6. *Opium for Diabetes.* Prof. Duchek. (*Wien. Med. Preise; Memorabilien*, xx. 7.)

The treatment of diabetes with opiates, introduced by M'Gregor, 1837, has yielded different results in the hand of different physicians. Prof. D. strongly asserts that opium, or morphine rather, exerts a direct influence upon the excretion of sugar, diminishing its quantity or making it disappear entirely. But large doses must be given, and to the administration of too small doses does he attribute the ill success of other practitioners. He bases these assertions on the clinical observations of fourteen cases, in which satisfactory results were obtained by the employment of large doses of morphine. "In most of the cases the disease had lasted a long while, and visibly influenced the nutrition of the patient. None of the patients were put on an absolute animal diet, but always a small amount of bread was allowed. In all cases a decrease in quantity of the sugar in the urine ensued, sooner or later, upon the administration of morphine, and if the dose was gradually increased, the excretion of sugar ceased temporarily. At the same time the excess of urine decreased, the thirst subsided, and the nutrition of the patient improved. With the most patients, it is true, this improvement lasted only as long as morphine was given; in one case, however, no sugar could be found in the urine eighteen months after the treatment had been stopped. The doses of morphine were gradually increased (to as much as three grains daily!) until the urine did not contain any sugar, then discontinued, to be given again when the sugar reappeared to the amount of two or three per cent., or moderate doses of morphine were continued, to keep the excretion of sugar below two per cent." The remedy is very well tolerated by such patients and its narcotic influence does not appear until very late, but it causes an obstinate constipation, which, however, can be counteracted from time to time by an enema or the use of rheum or aloe. Prof. D. does not feel prepared to assert that this treatment can cure every case of diabetes, but he thinks it may always lengthen the life of the patient.

7. *Treatment of Acute Articular Rheumatism with Starch Bandage.* FRANZ RIEGEL. (*Arch. f. Klin. Med.; Allg. Med. Centralz.*)

In 1845, Seutin and Gottschalk recommended the use of starch bandages for the treatment of acute articular rheumatism; the method, however, gaining but little credit with the profession, was soon forgotten. In 1871, Heubner revived the treatment, which he claimed had the advantages of lessening the pain, diminishing the fever and shortening the duration of the disease. The same good result was obtained in Wunderlich's clinic (Leipzig) where 45 cases had been submitted to the test of the starch bandage. Riegel tried a similar plan of treatment in the hospital of Cologne in a series of 41 cases of acute articular rheumatism. For the immobility of the affected joints, he used paste-board splints and sheet cotton; these materials making the simplest and cheapest bandage of the sort in question. The joint was wrapped in a thick layer of cotton; two splints previously softened in warm water, so as to be moulded by the form of the limb, were applied to each joint and secured in their places by a few turns of a roller bandage. There was no difficulty in getting complete immobility of the wrist, elbow, ankle and knee; but the shoulder and hip could not well be made quite immovable, and as a matter of course, the incomplete method of treatment yielded but an incomplete success in those joints. In order to have a fairer test, all internal medication was omitted in these cases. As to the results Riegel fully endorsed the assertion of others that the bandage quickly relieved the patient of all pain. "It is scarcely possible," he says, "to describe with words the wonderful change which takes place with the patient after the proper application of the bandage. The same patient who a minute ago screamed from pain and would not dare to move his limb, now allows the bandaged limb to be moved to and fro. And often the patients refuse to have the bandage taken off, for fear lest the pain will return." The bandage should not be removed until the soreness has left the joint for several days and the temperature has returned to the normal standard. The average time of retention of the bandage was six or seven days; the shortest was two days, and the longest a fortnight. In regard to the temperature, fever, and the duration of the disease, the beneficial influence of the bandage was confirmed by R., though he considered it as consequential; when the joints were placed under favorable conditions they at once began to improve, and as a simple consequence upon the decrease of local inflammation the febrile excitement abated and the whole course of the malady was curtailed. Among the 41 cases, there being seven which were complicated by endo- or pericarditis, R. concluded that the treatment by immovable bandages does not prevent these complications.

8. *Treatment of Cephalohæmatoma by Aspiration.* Gassner. (*Allg. Med. Centralz.*, September 15, 1875.)

A male infant, nine days old, was brought to Dr. G. on account of a large cephalohæmatoma, occupying the whole right parietal bone. Until then the tumor had been growing larger and tenser, and every other treatment having failed, he decided upon removing the blood by aspiration. The child lying on his back, the disinfected needle of the aspirator was introduced through the lower, *i. e.* posterior, part of the tumor into its cavity; an assistant holding the needle in a position parallel to the surface of the parietal bone, and exerting with the palm of his hand a gentle pressure on the tumor, the doctor withdrew by a slow and cautious aspiration, 100 grammes (over three ounces) of dark, liquid blood, and so thoroughly removed the tumefaction, that, at once, the soft tissues were in perfect apposition with the bone. The blood withdrawn, the needle of the aspirator proved the whole parietal bone denuded of its periosteum. During the operation the child was perfectly quiet, save when the needle was thrust through the scalp. The needle withdrawn, Lister's antiseptic dressing was applied, and a certain pressure exerted on the scalp by means of strips of adhesive plaster, to prevent a refilling of the tumor. On the ninth day after the operation, this dressing was removed for the first time, when everything was found in a normal condition.

9. *Gastro-Pulmonary Fistula Consecutive to Gastric Ulcer.* INLINSBERGER. (*Berl. Klin. Woch.*, No. 51; *La France Médic.*, October 2.)

Muco-purulent expectoration, mixed with alimentary substances, occurred in a patient whose epigastric uneasiness and distension were relieved by the ejection of these matters.

It was discovered that the pyloric extremity of the stomach had become adherent to the liver, at the insertion of its suspensory ligament. A large perforation of the pyloric portion of the stomach commmunicated above with a cavity as large as the fist, full of air and putrid matter. The parietes of the latter were formed by the thoracic walls, anteriorly and to the right; by the liver, below; by the stomach on the left; and posteriorly and inferiorly by the diaphragm. This muscle was adherent to the pleura and the lung, and exhibited an opening similar to that in the stomach. Into this cavity opened the bronchi, and thus was effected a communication with the trachea. There were several gastric tumors, some of which had ulcerated.

